# Fire Ants Protect Mealybugs against Their Natural Enemies by Utilizing the Leaf Shelters Constructed by the Leaf Roller *Sylepta derogata*

**DOI:** 10.1371/journal.pone.0049982

**Published:** 2012-11-21

**Authors:** Aiming Zhou, Ling Zeng, Yongyue Lu, Yijuan Xu, Guangwen Liang

**Affiliations:** 1 Red Imported Fire Ant Research Center, South China Agricultural University, Guangzhou, People’s Republic of China; 2 Hubei Insect Resources Utilization and Sustainable Pest Management Key Laboratory, College of Plant Science and Technology, Huazhong Agricultural University, Wuhan, People’s Republic of China; Université de Strasbourg, France

## Abstract

The importance of mutualism is receiving more attention in community ecology. In this study, the fire ant *Solenopsis invicta* was found to take advantage of the shelters constructed by the leaf roller *Sylepta derogata* to protect mealybugs (*Phenacoccus solenopsis*) against their natural enemies. This protective effect of fire ant tending on the survival of mealybugs in shelters was observed when enemies and leaf rollers were simultaneously present. Specifically, fire ants moved the mealybugs inside the shelters produced by *S. derogata* on enemy-infested plants. Compared with that in plants without ants, the survival of mealybugs in shelters in the presence of natural enemies in plants with ants markedly improved. Both the protection of ants and the shelters provided by leaf rollers did not affect the survival of mealybugs in the absence of enemies in plants. Ants and leaf rollers significantly improved the survival of mealybugs in predator-infested plants, whereas no such improvement was observed in parasitoid-infested ones.

## Introduction

Mutual interactions between hemipterans and invasive ants, which are known for their aggressiveness and high colony density, occur extensively in ecosystems [1]–[3]. The relationship between ants and hemipterans is characterized by the protection that ants provide hemipterans against predators and parasitoids [4], [5]. In return, the ants receive large amounts of honeydew, which is essential to their colony growth and survival because it contains sugars mixed with various amino acids [2], [6]–[9]. Previous studies have shown that the tending capability of ants improves the population growth of hemipterans by reducing not only predation and parasitism from natural enemies but also the risk of fungal infection [7], [10]–[14]. Fire ants are able to protect honeydew-producing hemipterans with their ability to deter natural enemies. The red imported fire ant *Solenopsis invicta* from the southeastern region of the United States has been reported to construct shelters for honeydew-producing hemipterans [2]. However, whether *S. invicta* protects hemipteran insects using shelters constructed by other species remains to be further explored.

*S. invicta* is a dangerous pest with a worldwide distribution, such as in the United States, Australia, New Zealand, and China [15], [16]. The negative effects of *S. invicta* on agriculture and forestry production, human health, and poultry production in South China have already been reported [17], [18]. *Phenacoccus solenopsis* Tinsley is native to the United States and has spread throughout the world, including Central America, South America, and Africa. It was reported to have invaded China and has shown potential damage to cotton [19], [20]. Mutual interactions mediated by honeydew occur commonly between hemipterans and invasive ants [1]–[3]. *P. solenopsis* produces large amounts of honeydew being consumed by fire ants [10], indicating a conditional mutualism between these two invasive species mediated by the sugar source [21]. *Sylepta derogata* (Fabricius) is a phytophagous lepidopteran that feeds on many host plants, such as *Hibiscus rosa-sinensis*, *Hibiscus syriacus*, *Gossypium hirsutum*, *Solanum melongena*, and *Vigna unguiculata* [22]. These pests are greatly populated and widely distributed in China, with *H. rosa-sinensis* and *G. hirsutum* being their favorite host plants [23]. We found that *H. rosa-sinensis* leaves were curled up by leaf roller larvae and served as pupation sites as well as that the curled leaves were probably utilized as shelters for mealybugs. Therefore, we hypothesized that fire ants protect mealybugs against their enemies using the leaf shelters constructed by *S. derogata*. We also verified whether the tending capability of ants and the shelters constructed by leaf rollers could enhance the survival of mealybugs on enemy-infected and uninfected plants. Our results provide new insights about the mutualism between invasive ants and honeydew-producing hemipterans and may contribute to improving this interaction for the successful invasion of these species.

## Materials and Methods

### Host Plants

*H. rosa-sinensis* was purchased from a commercial horticultural farm. All plants measured approximately 25–30 cm in height and were cultivated in plastic flower pots.

### Insects

All insects were collected from the suburbs of Guangzhou. *P. solenopsis* were fed on *H. rosa-sinensis* and maintained in the laboratory at 27±2°C and a relative humidity of 60%–70%. Each colony of *S. invicta* was divided into several subcolonies (∼1,000 workers and one queen) measured using a microbalance (Sartorius, BS, 224S). The ants were placed in a 9-cm plastic Petri dish with moistened plaster, which served as their artificial nest. They were given fresh, live *Tenebrio molitor* larvae and a 10% honey solution mixed with water (50 ml) weekly.

The lady beetle *Menochilus sexmaculata* and parasitoid *Aenasius bambawalei* are common enemy species of *P. solenopsis* in South China [24]. For this study, they were collected from *H. rosa-sinensis* plants in the field. *M. sexmaculata* were fed with mealybug nymphs in the laboratory, whereas *A. bambawalei* were initially collected as mummies (parasitoid pupae encased in dead aphids) and subsequently separated into gel capsules (∼10 mm in length) until adults emerged. Afterward, the wasps were classified according to sex and randomly paired. Copulation was observed in all pairings, and female wasps were used in experiments 24 h after the initial pairing.

### Field Investigation

This investigation was conducted in the experimental fields of South China Agricultural University. After ant colonies (∼50,000 workers and five queens) were transferred to the plot (2 m×6 m) edge and established, we planted four *H. rosa-sinensis* plants in a single file at distances of 0.8, 1.6, 2.4, and 3.2 m, respectively. Two leaf roller larvae were introduced to each *H. rosa-sinensis* plant, and they subsequently formed shelters. Mealybugs (120 per tube) were placed in a small plastic tube (1 cm×4 cm) and fixed on the epicormic branch of each *H. rosa-sinensis* plant. The mealybug larvae would climb out of the tube onto the leaves. These experiments were conducted using (1) mealybugs alone, (2) mealybugs with leaf rollers, (3) mealybugs with tending ants and leaf rollers, and (4) mealybugs with tending ants alone. A single *H. rosa-sinensis* was planted on an ant-free plot. [Plots without fire ant infestation were chosen to exclude colonies of *S. invicta*.] After 2 weeks, we counted and recorded the number of foraging ants on the plants, the numbers of mealybugs inside and outside the shelters, and the total number of mealybugs present on the plants. Each treatment was replicated eight times.

We conducted our field studies in areas where fire ants and mealybugs occur and for which no specific permits were required. The plots used were covered sparsely with weeds and seldom disturbed artificially before the experiments. The land used as the study area is neither privately owned nor protected in any way, and the field studies did not involve endangered or protected species.

### Laboratory Experiments

Two leaf roller larvae were transferred to *H. rosa-sinensis* leaves without mealybugs. When the leaf roller pupated, second instar mealybugs were transferred to the *H. rosa-sinensis* leaves (100 per plant). After 24 h, two lady beetle larvae and two parasitoid adults were separately placed on the plants. All plants were caged in wooden frames (60 cm×60 cm×40 cm) covered with nylon mesh. After another 24 h, the ant subcolonies were connected to their assigned plants through a plastic tube (1.5 cm in diameter). These experiments were conducted using (1) mealybugs alone, (2) mealybugs with leaf rollers, (3) mealybugs with predators and leaf rollers, (4) mealybugs with predators, (5) mealybugs with parasitoids and leaf rollers, and (6) mealybugs with parasitoids. We counted and recorded the numbers of surviving mealybugs on enemy-removed plants, on predator-infected plants, and in shelters after 1 week, whereas those of surviving mealybugs on parasitoid-present plants and in shelters were counted after 2 weeks. Each treatment without ant tending served as the control. All treatments were replicated 10 times.

### Statistical Analysis

After arcsine transformation, all data were tested for normal distribution using the Shapiro–Wilk test to compare differences in the survival of *P. solenopsis* between leaf roller-infected and uninfected plants, between predator- or parasitoid-infected and uninfected plants, and between plants with and those without ant tending. If the data were normally distributed, the *t* test was performed to compare means among all measured variables. The non-parametric Kruskal–Wallis test was used to compare the numbers of foraging ants present on plants placed at different distances from ant colonies. Linear regression was used to analyze the correlation between the number of surviving mealybugs in shelters and the number of foraging ants on plants. All statistical methods were conducted using SPSS 14.0.

## Results

### Effects of Ant Tending and the Shelters on the Survival of Mealybugs in the Field

**Figure 1:**
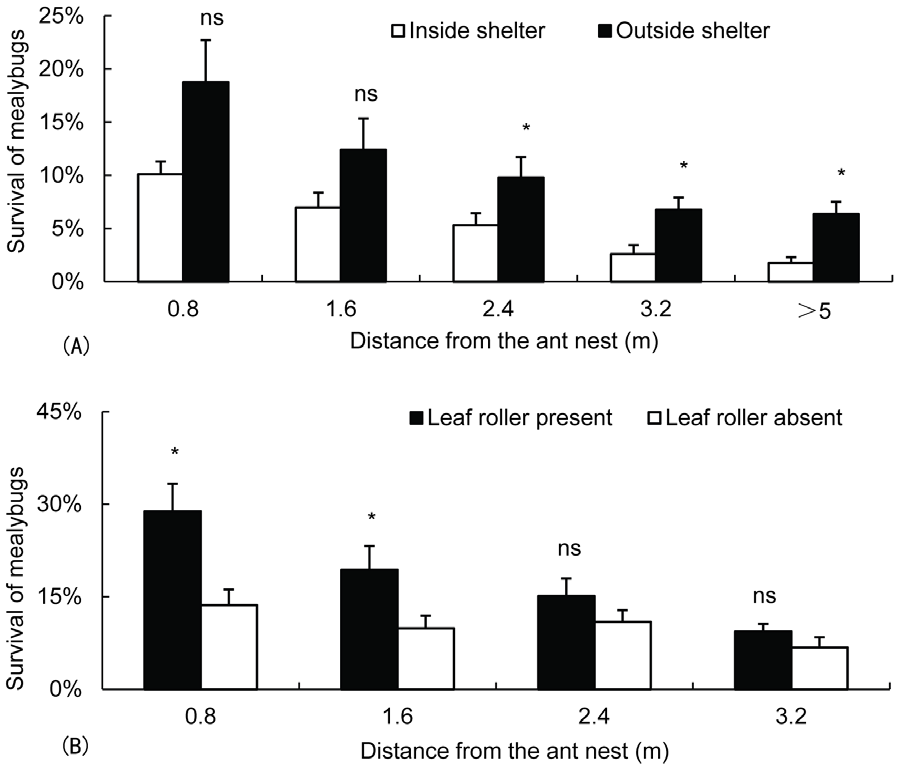
Survival (mean±SE) of mealybugs in the field (A) between inside and outside shelters as well as (B) between plants with and those without shelters. “ns” indicates not significant (*p*>0.05), whereas the asterisk indicates significant difference (*p*<0.05) in the number of mealybugs present between treatments (paired *t* test).

**Figure 2:**
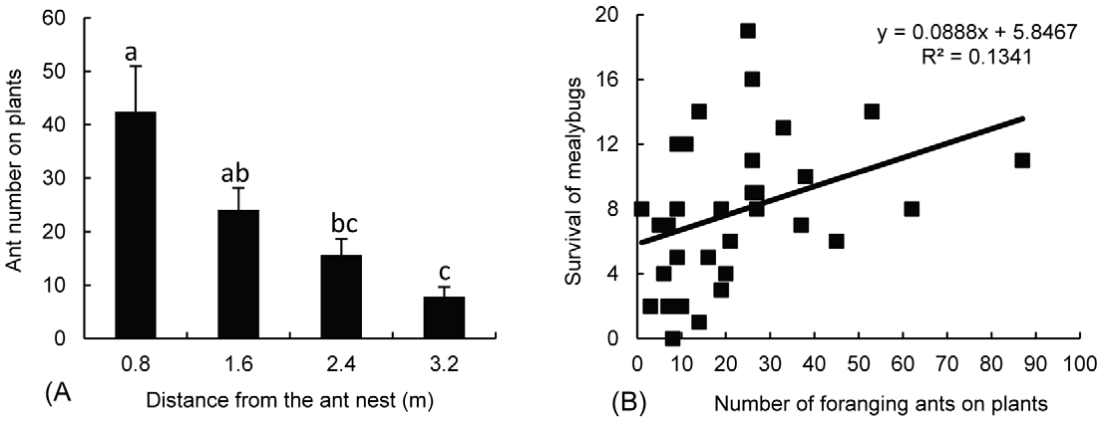
(A) Number of foraging workers (mean±SE) of *S. invicta* at different distances from the ant colonies and (B) its correlation with the number of mealybugs present in shelters. Bars labeled with the same letter are not significantly different from each other (*p*>0.05, Mann–Whitney test).

We found that ant workers occurred in the shelters and gradually transported or drove mealybugs toward them when their natural enemies were present. Fire ants increased the survival of mealybugs, although predators and parasitoids could enter the shelters. In the field, no significant difference between surviving mealybugs inside the shelters and those surviving outside was detected when the plants were close to an ant colony (0.8 m: *t* = −2.294, *df* = 7, *p* = 0.056; 1.6 m: *t* = −2.141, *df* = 7, *p* = 0.069) (Fig. 1A). However, a significant difference was observed when the plants were distant from an ant colony (2.4 m: *t* = −3.330, *df* = 7, *p* = 0.013; 3.2 m: *t* = −2.620, *df* = 7, *p* = 0.034; >5 m: *t* = −3.582, *df* = 7, *p* = 0.009) (Fig. 1A). When the plants were only 0.8 and 1.6 m away from the ants’ nest, the total number of surviving mealybugs in sheltered plants was significantly higher than that in unsheltered ones (*t* = 2.964, *df* = 14, *p* = 0.010; *t* = 2.169, *df* = 14, *p* = 0.048). However, no significant difference was observed when the plants were 2.4 and 3.2 m away from the ants’ nest (*t* = 1.218, *df* = 14, *p* = 0.243; *t* = 1.268, *df* = 14, *p* = 0.226) (Fig. 1B). In addition, fewer ants were found when plants were placed at a greater distance from ant colonies (Kruskal–Wallis test: *χ* ^2^ = 16.796, *df* = 3, *p* = 0.001) (Fig. 2A). The number of surviving mealybugs in shelters was significantly linearly (*y* = 0.089*x*+5.847; *F* = 4.647, *p* = 0.039) correlated with the number of foraging ants on a given plant (Fig. 2B).

### Effects of Ant Tending and the Shelters on the Survival of Mealybugs on Enemy-infested Plants

**Figure 3:**
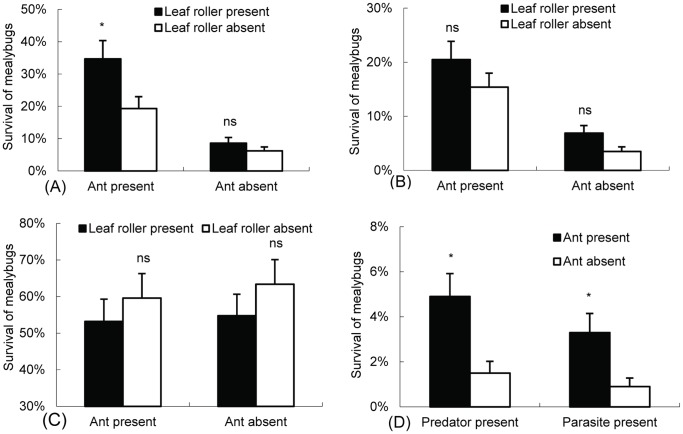
Survival (mean±SE) of mealybugs under laboratory conditions (A) on predator-infected plants, (B) on parasitoid-infected plants, (C) on enemy-removed plants, and (D) in shelters on enemy- and leaf roller-infected plants. “ns” indicates not significant (*p*>0.05), whereas the asterisk indicates significant difference (*p*<0.05) in the number of mealybugs present between each treatment (*t* test).

When lady beetle larvae were present and the ant colony had access to the plants, the survival of mealybugs on plants with leaf rollers was remarkably greater than that of mealybugs on plants without leaf rollers (*t* = 2.765, *df* = 9, *p* = 0.022) (Fig. 3A). However, no significant difference was found between plants with and those without leaf rollers when the ant colonies were separated from them (*t* = 1.090, *df* = 9, *p* = 0.304) (Fig. 3A).

When parasitic wasps were present, no apparent difference was observed in the survival of mealybugs between plants with and those without leaf rollers, regardless of whether or not the ant colonies had access to the plants (*t* = 1.443, *df* = 9, *p* = 0.183; *t* = 1.620, *df* = 9, *p* = 0.140) (Fig. 3B).

### Effects of Ant Tending and the Shelters on the Survival of Mealybugs on Enemy-excluded Plants

When natural enemies were removed from the plants, no significant difference was found in the survival of mealybugs between plants with and those without leaf rollers when the ant colonies had access to the plants (*t* = −0.663, *df* = 9, *p* = 0.524) (Fig. 3C). The situation in plants without an ant colony was similar to that in plants with one (*t* = −1.042, *df* = 9, *p* = 0.325) (Fig. 3C).

### Effects of Ant Tending on the Survival of Mealybugs in Shelters on Enemy-infested Plants

When lady beetle larvae were present on the plants, the survival of mealybugs in shelters with an ant colony was significantly greater than that of mealybugs on plants without an ant colony (*t* = 2.782, *df* = 9, *p* = 0.021) (Fig. 3D). When parasitic wasps were present on the plants, a significant difference in the survival of mealybugs was found between plants with and those without an ant colony (*t* = 2.295, *df* = 9, *p* = 0.047) (Fig. 3D).

## Discussion

Previous studies have indicated that *S. invicta* constructs shelters for hemipterans to avoid parasitic enemies [2]. Our study is the first to report that fire ants used the shelters constructed by leaf rollers to protect their mealybug mutualists. We verified this interesting interaction in the field and under laboratory conditions. We also found that mealybugs had difficulty entering the shelters successfully without the assistance of fire ants, demonstrating that the co-occurrence of ant tending and the shelters constructed by leaf rollers improved their survival in shelters on predator-infested plants. Shelters also enabled ants to monopolize the honeydew produced by the hemipterans. Our results indicate that the mutualism between fire ants and mealybugs facilitates the fitness of both species through their interaction [7], [13].

We have confirmed that mealybugs benefit from the above-described interaction because their survival in shelters was significantly higher than that of mealybugs on plants with an ant colony (Fig. 3D). The results demonstrated that the ants transferred mealybugs into the shelters produced by leaf rollers (Fig. 1), in agreement with research showing that *S. invicta* actively moves homopterans between plants to avoid predators and parasitoids [25]. In addition, our results indicate that ant tending and the shelters constructed by leaf rollers could not significantly improve the survival of mealybugs on parasitoid-infested plants (Fig. 3B). We therefore concluded that ants still move mealybugs toward the shelters on plants with parasitoids but that parasitic wasps find them and lay eggs on their body. This conclusion was supported by the number of mealybug mummies determined in the shelters at the end of the experiments.

In summary, mealybugs benefit from their interaction with fire ants and *S. invicta* ants recognize the situation of mealybugs and respond accordingly. Although *S. invicta* is considered an omnivorous species and colonies of fire ants can survive without honeydew-producing insects, this study confirms that mealybug husbandry is beneficial in that ants may evolve and develop defenses against their natural enemies and could be an important mechanism for establishing and spreading invasions.
